# Smart retail SKUs checkout using improved residual network

**DOI:** 10.1038/s41598-023-49543-x

**Published:** 2023-12-15

**Authors:** Chunchieh Wang, Chengwei Huang, Xiaoming Zhu, Zhi Li, Liye Zhao

**Affiliations:** 1 School of Instrument Science and Engineering, Southeast University, Nanjing, 210000 China; 2 Zhejiang Laboratory, Hangzhou, 310000 China; 3 School of Electrical Engineering, Southeast University, Nanjing, 210000 China

**Keywords:** Computer science, Electrical and electronic engineering

## Abstract

Intelligent signal processing in unmanned stores enhances operational efficiency, notably through automated SKUs (Stock Keeping Units) recognition, which expedites customer checkout. Distinguishing itself from generic detection algorithms, the retail product detection algorithm addresses challenges like densely arranged items, varying scales, large quantities, and product similarities. To mitigate these challenges, firstly we propose a novel boundary regression neural network architecture, which enhances the detection of bounding box in dense arrangement, minimizing computational costs and parameter sizes. Secondly, we propose a novel loss function for hierarchical detection, addressing imbalances in positive and negative samples. Thirdly, we enhance the conventional non-maximum suppression (NMS) with weighted non-maximum suppression (WNMS), tying NMS ranking scores to candidate box accuracy. Experimental results on SKU-110K and RPC datasets, two public available databases, show that the proposed SKUs recognition algorithm provides improved reliablity and efficiency over existing methods.

## Introduction

Objects within visual signals can be quantified, identified, and analyzed utilizing interdisciplinary approaches, such as digital signal processing and machine learning algorithms^1–3^. Effectively processing visual information, including tasks like object counting, target size measurement, and object attribute classification, necessitates the use of high-quality sensors and precise instrumental components^4,5^. With the advancement of deep neural networks, emerging algorithms exhibit an increased ability to accommodate a broader spectrum of hardware configurations, subsequently enhancing measurement accuracy^6,7^.

The advancement of digital signal processing capabilities and the evolution of smart sensors have enriched the concepts of new retail, such as unmanned stores and automated cash registers. A substantial number of supermarkets and retail outlets have adopted self-service cashiers for transaction processing. A prominent requirement in the realm of new retail is the swift and accurate detection of retail Stock Keeping Units (SKUs), which gives rise to a pivotal avenue of future research.

Traditional approaches for digitizing retail products involve the utilization of barcode scanners and RFID scanners. The automation of product checkout in supermarkets predominantly relies on scanning barcode labels or employing RFID tags for inductive identification. Despite their widespread use, barcode-based methods are notably inefficient, as they necessitate manual scanning of each individual barcode by consumers. Conversely, RFID-based techniques face challenges in large-scale implementation due to the relatively high cost of tag units. Therefore, exploring avenues to detect and identify product targets based on photographs taken by standard cameras holds significance.

Additionally, cameras and other visual sensors can capture more comprehensive information about retail products. Detectors based on deep learning algorithms can swiftly and dependably identify numerous objects within real-world scenes. These methods have found widespread practical application. For example, RetinaNet^8^ is a famous object detection network, of which backbone is consist of ResNet^9^ and FPN^10^. Four different sizes of feature pyramid can be extracted after putting the input image into the backbone network. Subsequent to obtaining feature pyramid information, the process involves employing a classification network and a bounding box generation network for each individual layer within the feature pyramid. These two sub-networks are modified from RPN^11^ network, and the anchors are used to create a series of candidate areas (proposals). Unlike the Region Proposal Network (RPN), which can solely differentiate between foreground and background objects, the classification network within RetinaNet has the capability to directly discern between different categories present within the dataset. For each layer of the feature pyramid, predictions regarding object categories and their corresponding positions are generated and subsequently fused together. Detecting the category and precise position of the object are pivotal objectives in retail object detection.

Subsequently, the process involves employing Non-maximum Suppression (NMS) to yield the final detection outcomes. Non-maximum Suppression serves as a post-processing step in the majority of object detection pipelines. While it is customary to apply suppression to refine detection outcomes, this suppression technique can be enhanced through a novel weighted approach, as elaborated in section. Despite the remarkable advancements made in object detection algorithms in recent times, even the most advanced general object detectors tend to exhibit suboptimal performance when applied to the task of detecting retail products in real-world scenarios. While these methods excel in tasks such as general object detection, vehicle detection, and pedestrian detection, they often encounter challenges when dealing with densely arranged retail products on shelves or products arranged disorderly on cashier desks. This can lead to a range of issues, including low recall rates, significant occurrences of repeated detection, and imprecise bounding box delineations.

Based on the analysis of retail product images in real scenes, we believe that dense retail product detection is encountering the following challenges: (1) Objects of similar categories are densely clustered and possess identical characteristics, making it difficult for the detector to distinguish between individual objects; (2) Multiple targets often become covered or overlapped, posing challenges for the detector to accurately discern the boundaries of individual objects; (3) The considerable number of objects, coupled with their small sizes, further complicates the detection process. Addressing these challenges is crucial for enhancing the accuracy and effectiveness of dense retail product detection. conventional classifiers are limited by grid cells, so it is difficult to recognize the targets accurately. (4) Post-processing mechanisms such as NMS are likely to incorrectly filter out the correct results by mistake.

Frequently, existing retail product detection methods employ clustering algorithms to generate anchor frames of various scales and navigate each detection area individually. According to the anchors of multiple scales^11^, these methods generate (X, Y, W, H) coordinates and the confidence of the proposal frame. However, the approach of generating the coordinates of the four corner points of a candidate frame based on anchor points is not entirely suitable for product detection scenarios. This is due to the fact that SKUs of similar categories situated on the same shelves often share a similar shape and are closely arranged. In scenarios involving check-out settlements, a wide array of SKUs with distinct shapes are present, posing challenges for anchor boxes to accurately capture the appropriate parameters.

**Figure 1:**
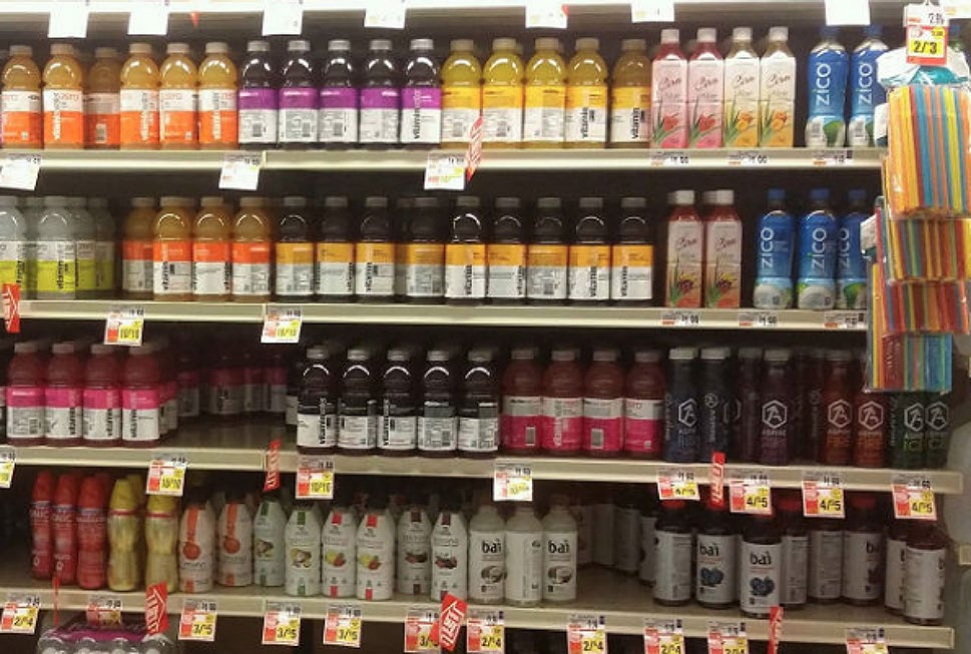
Example of dense display of retail products.

In the detection of similar categories of SKUs on the same shelf, as shown in Fig. 1. The goods possess identical sizes, leading to the utilization of a single detection box across a vast scene area. Consequently, this approach significantly reduces the recall rate. Furthermore, numerous factors can impact the model’s outcomes, particularly the count of anchor frames, which necessitates calibration within the hyperparameters. Challenges arise in scenarios involving small targets, primarily due to the constrained number of anchor frames, thereby limiting the model’s generalization capability. When dealing with densely arranged products, a notable proportion of anchor frames fall into the negative sample category, introducing an imbalance within the sample distribution and subsequently increasing computational costs.

To overcome these challenges, we propose implementing a Boundary Regression Network (BRN). This approach departs from the original prediction method that relies on multiple anchors and instead directly engages in boundary regression for product objects within specific regions across various scales. Once the center point of the candidate region is established, determining the position and shape of the candidate box becomes attainable by regressing the distances between the four edges of the boundary box and the center point. This method not only reduces the number of hyperparameters introduced by anchors but also eliminates the need for calculating Intersection-over-Union (IoU) between all anchors and real objects, thereby significantly minimizing parameters and computational demands. Moreover, by capitalizing on the substantial number of negative samples generated by the preceding anchor-based algorithm, BRN promotes a more balanced distribution of samples during the training process. This innovative approach contributes to a comprehensive enhancement in the efficiency and efficacy of the detection model.

**Figure 2:**
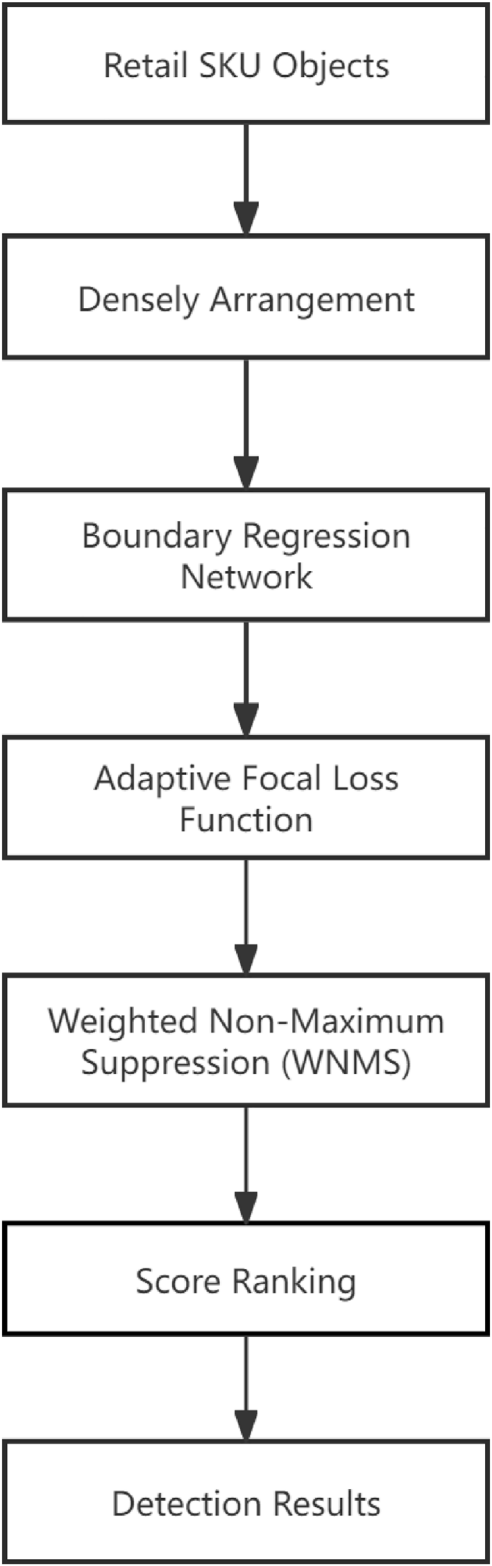
Flowchart of the proposed dense object detection based on boundary regression network.

The overall flowchart of the dense object detection is shown in Fig. 2, which includes the dense scene, network module, the loss function, the weighted NMS module and the detection advantages. Results on typical datasets show that the proposed method is effective in identifying dense commodities, and performs better on mAP than the commonly used target detection algorithms in industry.

## Related work

Various visual algorithms may be used for smart sensors and cameras^12^. In contrast to barcode scanners, visual detection of retail objects offers a broader spectrum of information, rendering it better suited for fulfilling the demands of automated checkout processes. Hofman et al.^13^ introduced X-Detect, an adversarial patch detector for object detection models used in domains like retail. X-Detect achieved real-time detection of adversarial samples, provided explanations for alerts, and effectively handled unfamiliar threats. Dhonde et al.^14^, proposed methodology involved using pretrained models to compute an adaptive Region of Interest, and utilizing a custom-trained model to identify and track products in video frames. Pan et al.^15^ also studied densely placed object detection. However, it is not specifically designed for boundary regression, resulting in less-than-ideal detection of object boundaries. In contrast, our approach utilizes regression to identify accurate boundaries, a crucial aspect for SKU detection, especially considering that goods are often densely overlapped on shelves. Another drawback of their work is its limited ability to model subtle differences between objects. In contrast, our model addresses a common challenge in SKU detection, where goods’ packaging and appearances can be highly similar to each other.

Visual feature analysis can be approached in two ways. Qiu et al.^16^ studied fire detection using sensors combined with image processing modules. Fire and flames were treated as visual targets, and the approach involved employing hand-crafted features such as edges, rather than relying on representation learning techniques. On the other hand, Wei et al.^17^, used end-to-end representation learning approach to solve image classification problems, which was superior than hand-crafted approaches in their applications.

Prior method of camera-based retail product detection was mainly based on traditional manual feature extraction, such as SIFT^18^ and HOG^19^ etc. The Histogram of Oriented Gradients (HOG) technique has found extensive application in addressing object detection challenges. It helps balance feature invariance, encompassing factors like translation, scale, and illumination, across various object classes, thereby enhancing nonlinearity in distinguishing between different object categories. The Deformable Part Model (DPM)^20^ builds upon and extends the HOG algorithm. It encompasses a primary filter alongside multiple secondary filters. By incorporating boundary regression and context priming techniques, DPM enhances detection accuracy. Regarded as a leading traditional detection algorithm, DPM operates swiftly and can accommodate object deformations. However, its stability falters when faced with substantial rotations. Presently, features computed through conventional methods fall short of capturing the intricate semantic information embedded within images. Consequently, these methods lack stability in various scenarios.

Presently, there exist two primary paradigms for object detection grounded in deep learning: the first approach entails a two-stage detection algorithm that incorporates a region proposal network. In this method, proposal regions are obtained and subsequently classified. A notable example is the R-CNN series^21^. The second approach involves a one-stage detection algorithm that directly eliminates the need for autonomous proposal region search. An exemplar is the Single Shot MultiBox Detector (SSD)^22^. Of the two methods mentioned, the two-stage approach with a region proposal has demonstrated superiority in detection and positioning accuracy. Conversely, the one-stage end-to-end algorithm shines in terms of speed. However, with the ongoing depth of research and technological advancements, the accuracy of one-stage algorithms has seen significant enhancements, surpassing even the performance of two-stage algorithms reliant on proposal boxes. This progress makes one-stage algorithms well-equipped to handle most daily tasks. Moreover, their faster operational speed accentuates their advantages over two-stage algorithms such as Faster R-CNN^11^.

## Methodology

### Over-all model architecture

**Figure 3:**
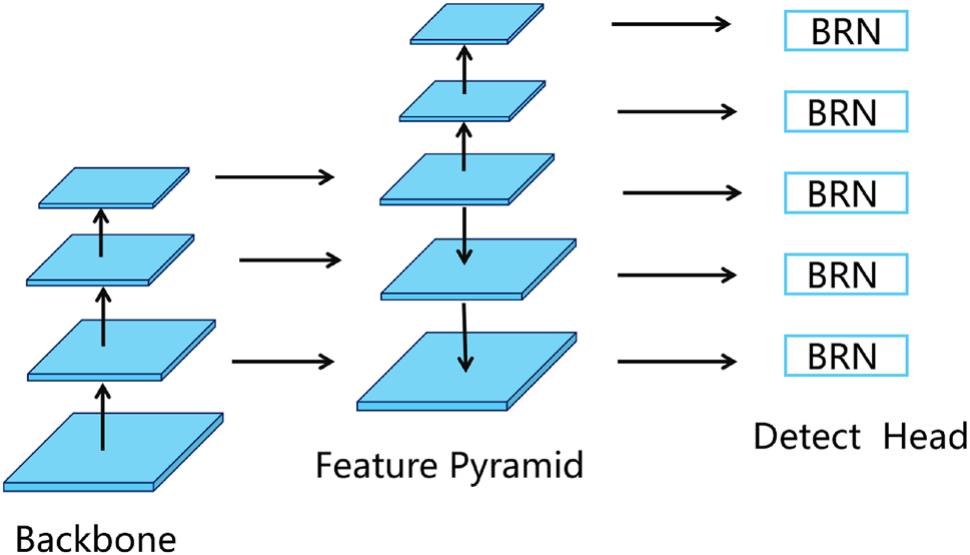
The over-all network architecture of object detection.

In this section, we treat the retail SKU recognition as a regression problem and propose a novel boundary regression neural network to solve it. In order to explore the limit of detection accuracy in the scenario of dense shelves, we divide the overall network into three parts: feature extraction layer, feature fusion layer and detection layer, as shown in Fig. 3. Firstly, the feature extraction module is the backbone network of the whole target detector, such as VGG^23^, Darknet^24^, etc. Due to the flexibility and variability of network depth, the classic network ResNet^9^ is selected as the backbone network in this paper. Feature fusion modules are often composed of feature pyramid networks, such as Bi-FPN and PANet^25^, etc. In this paper, the classic network FPN^10^ is also used to achieve multi-scale image feature fusion. The detection layer is different from other existing models. In response to the unique characteristics of densely arranged, numerous, and visually similar SKUs, we have developed a distinct boundary regression network.

**Figure 4:**
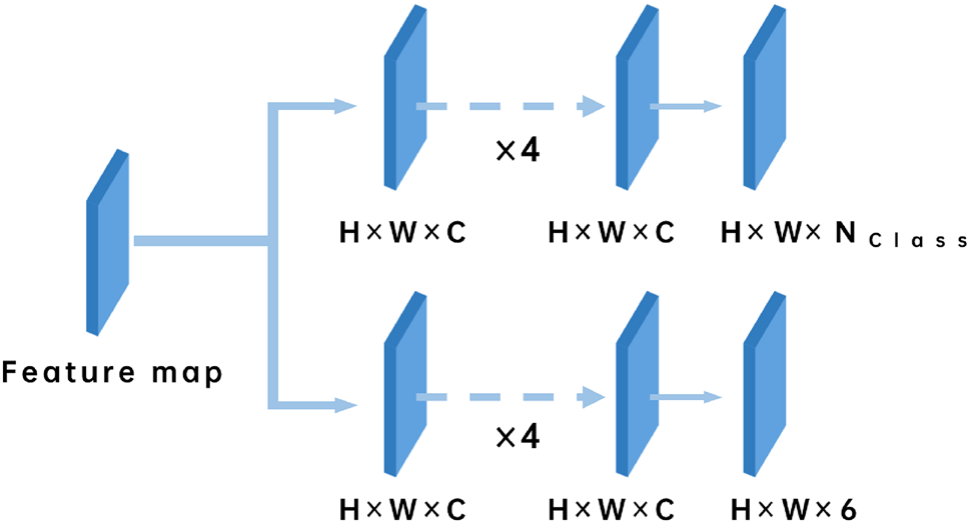
The proposed network architecture of boundary regression network.

Most of the general object detection algorithms are based on the pre-calculated anchor boxes to predict the bounding boxes, but this kind of method is not suitable in the case of a large number of SKUs and dense distribution. Within this paper, we treat object detection as a regression challenge, and we introduce the Boundary Regression Network (BRN) to effectively address the issue of densely positioned products. As shown in Fig. 4, the Feature Map obtained from the preceding feature fusion layer undergoes multiple convolutional layers to alter the number of channels. $N_{class}$ stands for the number of classes. This process facilitates the regression of the boundary position for the targeted product.

*H* and *W* here denote that the original map is divided into $H \times W$ regions. Every region assumes responsibility for detecting the nearest center point to its own center point. The detection range encompasses the product object within the respective region, as shown in Fig. 4. The feature map corresponds to the position of the original sample one by one.

**Figure 5:**
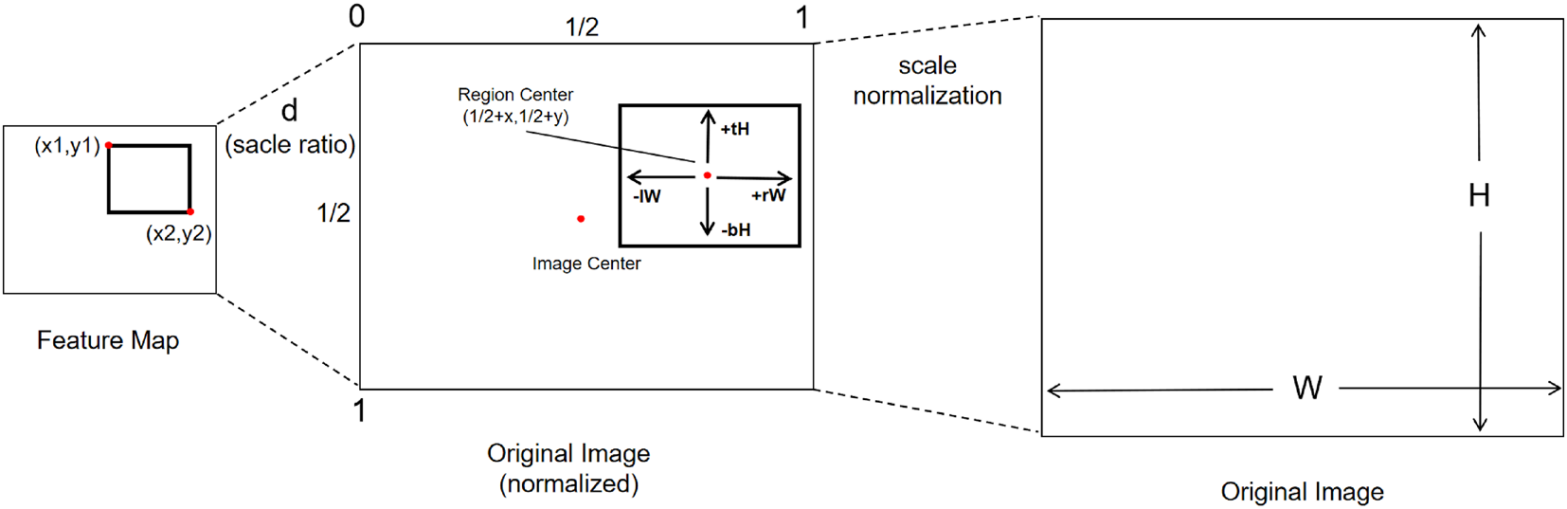
Geometrical illustration of coordiates mapping.

The geometrical illustration is depicted in Fig. 5. *d* is a scale ratio between the feature map scale and the original image scale, the central point coordinate corresponding to a certain coordinate (*x*, *y*) in the feature graph is $\left( {dx + \frac{d}{2},dy + \frac{d}{2}} \right)$. In the presence of an object within the current region, the Boundary Regression Network (BRN) calculates the distances between the boundaries encompassing the object and the center point of the current detection region, denoted as (*l*, *t*, *r*, *b*), and these four values respectively represent the distance between the center point of the current region.These four values can be translated and transformed into the coordinates of the actual target’s four corners. The conversion of the regression values from BRN to the coordinates of the four corners can be expressed in Eq. (1).

1 $$\begin{aligned} \begin{aligned} x_1&= \frac{d}{2}+dx - Wd \times l \\ y_1&= \frac{d}{2} +dy - Hd \times t \\ x_2&= \frac{d}{2} + dx + Wd \times r \\ y_2&= \frac{d}{2} + dy + Hd \times b \end{aligned} \end{aligned}$$

The first multi-layer convolution of BRN gets a feature vector of $(H \times W \times 6)$, where the length of the channel vector is 6. Among the channel vector members, in addition to the above four distance values (*l*, *t*, *r*, *b*), there are two values representing prediction confidence *p* and prediction *score* respectively. Here, the predicted value *p* is a real number between (0, 1). When the four distance values are correctly predicted comparing to the ground truth of the target, ideally the confidence value is 1. When the four distance values are incorrectly predicted, the lower boundary of the confidence value is 0. In the supervised training, the real value 1.0 and real value 0.0 are used as the ideal value for calculating losses. The calculation method of prediction *score* will be introduced in detail in section NMS-Score. Another multi-layer convolution of BRN also obtains a set of feature vectors which represent the categories corresponding to the object of the current bounding box, as shown in Fig. 4.

In object detection tasks, it is plausible that the detection area associated with a specific feature map might encompass two or more actual target objects simultaneously. While conventional methods like Faster R-CNN can identify multiple objects within a single detection area due to the utilization of various-shaped anchors, our BRN introduces a distinct approach. In cases where actual boundaries overlap, our BRN mandates that a detection area exclusively identifies the object closest to its center. This stems from the observation that in scenarios involving product identification, whether it’s product images on shelves or during cashier settlement, the center points of two objects cannot entirely coincide. This diverges from the common target detection scenarios in real-world settings.

Furthermore, the existence of multi-scale fusion networks like FPN and PAN results in the back-end BRN being constructed atop feature maps of varying scales. As a result, even if the center points of two genuine boxes are closely situated, a more detailed feature map is tasked with detection to effectively differentiate between them. This configuration substantially diminishes the count of network parameters, rendering the model simpler and more amenable to training. Additionally, it significantly alleviates the computational burden during the subsequent Non-maximum Suppression (NMS) process.

### The proposed loss function

The accuracy of one-stage object detection usually experiences a slight decrease compared to that of two-stage object detection, often attributable to class imbalance. In this context, the term “category” does not refer to the categories of SKUs but rather to the binary distinction between true and false instances. This classification relates to whether the currently predicted bounding box corresponds to a true positive or a true negative outcome.

**Figure 6:**
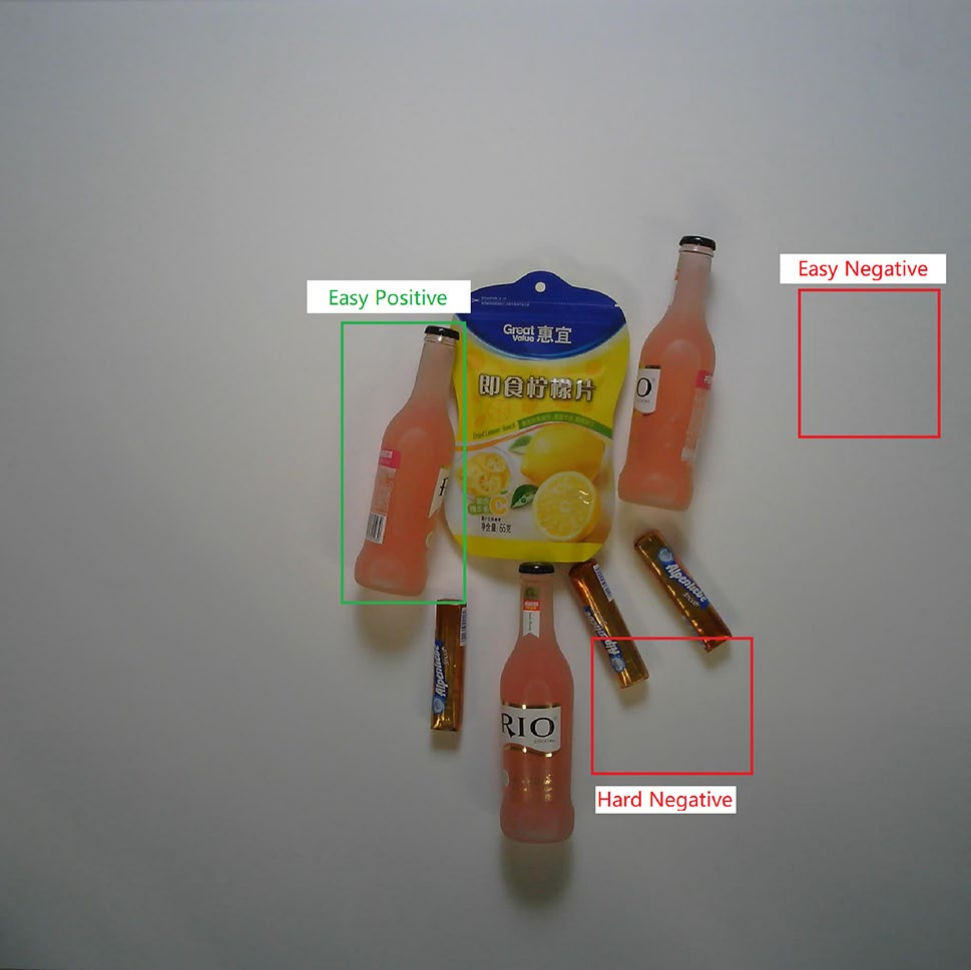
Depiction of “easy” and “hard”, positive and negative samples.

As shown in Fig. 6, due to the imbalance of positive and negative samples in the data set and too many negative examples, the loss value calculated by the loss function is too large and the loss contributed by the positive instances will be submerged, which causes difficulties in model converge.

Additionally, the degrees of difficulty in discriminating whether the bounding box is true or false vary. For example, when considering a particular bounding box situated in the background, the detector can easily identify it as false. When the bounding box is close to the actual target, or partially covered by one or more of the real objects, the result should be false. However, it is very difficult for the detector to identify accurately. In this case, the loss should be amplified so that the detector can better learn difficult tasks. At the same time, based on the multi-scale detection brought by FPN, we apply the boundary regression network to each scale of FPN output, so as to solve the problems of fuzziness and low recall caused by boundary overlap.

Therefore, an imbalance between positive and negative samples, along with varying degrees of difficulty, causes the loss function to fail in effectively guiding the training process towards the global optimal value. To tackle this issue, the introduction of a weighting mechanism becomes essential for distinguishing between different types of losses.

Let’s denote weight $\alpha$ and weight $\gamma$ for the loss function. Denote *p* as the predicted value of confidence. Building upon the concept of Focal Loss^8^, as shown in Eq. (2), we have restructur the binary cross-entropy loss function.

2 $$\begin{aligned} \text {FL} (p) = -\alpha \cdot (1 - p)^\gamma \cdot \log (p) \end{aligned}$$

Let’s denote $\omega$ as the distance between the center of prediction box and the center of real box. *c* is the class label indicating positive or negative samples.

We incorporate the weight coefficients and distance coefficients, and propose the Adaptive Focal Loss (AFL), as shown in Eq. (3).

3 $$ AFL(p,y) = \left\{ {\begin{array}{*{20}l}    { - \frac{\alpha }{{\omega  + 1}}(1 - p)^{\gamma } log(p),} \hfill & {y = 1} \hfill  \\    { - \frac{{1 - \alpha }}{{\omega  + 1}}p^{\gamma } log(1 - p),} \hfill & {otherwise} \hfill  \\   \end{array} } \right. $$

The weight $\alpha$ is used to balance the imbalance of positive and negative samples, and the weight $\gamma$ is used to distinguish the rate of weight reduction of difficult and easy samples and conditional simple samples. For the cross entropy loss function, $\gamma$ is 0. As $\gamma$ increased, the adjustment factor also increased, that is, the loss produced by simple samples was gradually inhibited. The reason for this effect is that for simple samples, the predicted value *p* of the positive sample tends to approach 1, while the predicted value *p* of the negative sample tends to approach 0. As the value of $\gamma$ increases, the loss generated by the simple sample decreases exponentially. In the face of different training sets, $\alpha$ and $\gamma$, as hyperparameters, need to be determined by multiple experiments.

### The proposed scoring mechanism

Whether employing a one-stage or two-stage object detection algorithm, the resulting predictions frequently yield numerous redundant bounding boxes. This redundancy originates from the possibility of the same object within the image being detected by prediction regions at different scales and positions. As a result, the utilization of a non-maximum suppression (NMS) algorithm becomes imperative to remove excessive bounding boxes, retaining only the most relevant ones. The majority of NMS techniques directly leverage Intersection-over-Union (IOU) thresholds for assessment^18^. Subsequently, based on confidence scores, the bounding boxes are ranked, with the highest-confidence predictions being retained.

However, this approach might not provide the most optimal solution. A high confidence level does not necessarily ensure the best alignment of the bounding box with the ground truth. In simpler terms, the confidence levels of positive and negative samples may not accurately reflect the positional accuracy of the bounding box. Therefore, it is imperative to redesign the scoring mechanism for the non-maximum suppression.

The proposed scoring mechanism, referred to as NMS-Score, concurrently function as a value within the regression result vector and establish the basis for NMS ordering. To address this issue, IoU-Net^26^ has been introduced as a potential solution. This method introduces an IoU branch between the predicted bounding box and the actual object, utilizing position confidence to replace category confidence. Building upon the aforementioned approach, this paper devises an NMS-Score based on the (*l*, *t*, *r*, *b*) distance information generated by BRN, as shown in Eq. (4). Let’s denote: $\Delta$ as the operator for calculating the *L*1 distance between the predicted value $(\hat{l},\hat{t},\hat{r},\hat{b})$ and the true value of (*l*, *t*, *r*, *b*), and $\varepsilon$ as a positive minimum value to prevent the denominator from being 0.

4 $$\begin{aligned} Score = tanh\left( \frac{1}{\Delta l + \Delta t + \Delta b + \Delta r + \varepsilon }\right) \end{aligned}$$

Thus, as the network-generated values (*l*, *t*, *r*, *b*) approach the true values, the parameters within the tanh function tend towards infinity, resulting in an NMS-Score of 1. Conversely, if the bounding box significantly deviates from the true value, the NMS-Score approaches 0. NMS-Score, distinct from mere confidence values, can be employed to filter out candidate boxes that closely align with the actual box. The Score value, acquired through deep neural network learning, encodes the positional accuracy information of the bounding box.

Therefore, when compared to the traditional NMS technique, utilizing the Score value as opposed to the simple confidence value is more appropriate as a ranking indicator for non-maximum suppression.

## Experimental results

### Dataset

Recognizing objects in a retail setting entails grappling with distinctive challenges. A significant hurdle arises in densely arranged scenes where objects are frequently obstructed, either partially or fully, by other items, shelves, or customers. This complexity renders accurate identification and classification a daunting task. The crowded nature of these environments further adds to the intricacy of the recognition process, demanding systems to adeptly distinguish between closely packed objects.

Another noteworthy challenge emerges from the high similarity between retail products. Items often exhibit shared shapes and colors, necessitating a recognition system endowed with a high level of discriminative capability. Fine-grained recognition models become pivotal in capturing subtle variations and distinguishing branding differences among visually similar items.

Additionally, the dynamic retail landscape introduces the challenge of frequent goods replacement, requiring recognition systems to be adaptable to changes in the types and positions of products. Furthermore, these systems must showcase the ability to rapidly learn from new data, all while maintaining a relatively low computational cost. This adaptability is crucial in ensuring the effectiveness of the recognition system in the face of ever-changing retail scenarios.

To address these challenges in smart retail, we adopt two public available benchmark test datasets: SKU-110K^27^ and RPC^28^. The SKU-110K dataset provides 11,762 images, which contain more than 1.7 million annotated bounding boxes captured in dense scenes. The dataset includes 8233 images for training, 588 validation set images and 2941 test set images, for a total of approximately 1,733,678 instances. The images were collected from thousands of supermarket stores, with varying proportions, viewing angles, lighting conditions and noise levels. All images are tuned to a megapixel resolution. Most instances in the dataset are tightly packed and object orientation is within the range of $[-15^{\circ },15^{\circ }]$.

SKU-110k data has the following characteristics: (1) the original image has a low resolution and a wide range of changes; (2) The difference between categories is small, and the SKUs on the same shelf are often similar in shape or color characteristics; (3) Products are very densely packed, with most images containing hundreds of objects.

**Figure 7:**
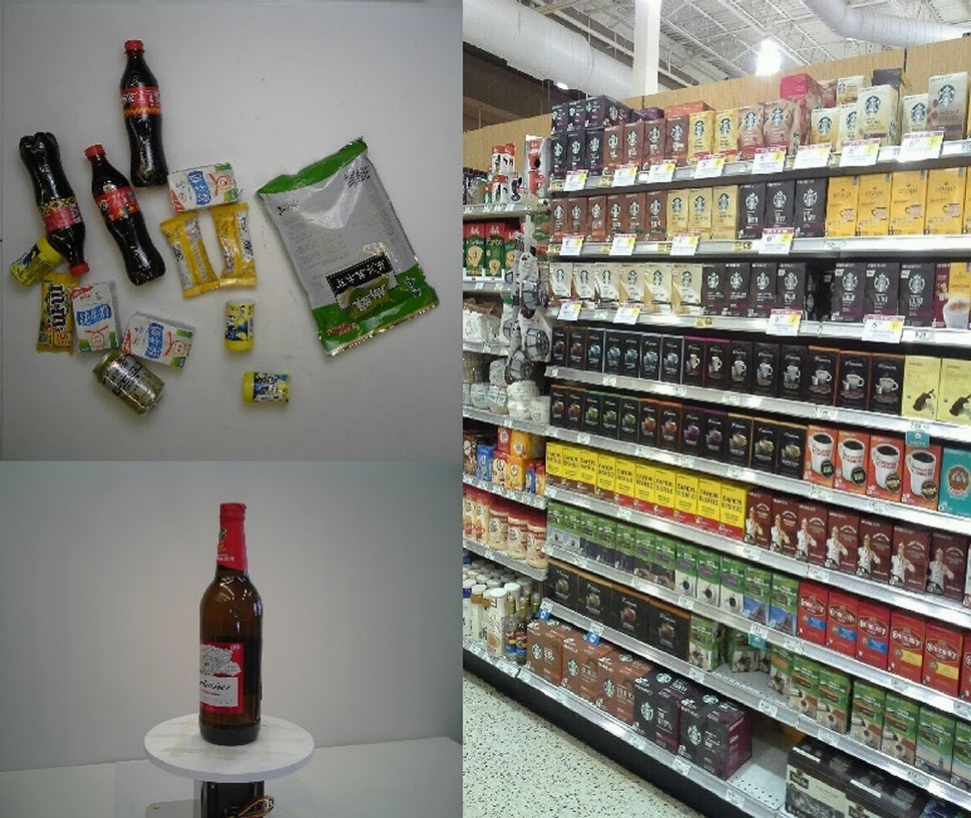
Dataset examples: RPC dataset (left), SKU-110K dataset (right).

RPC dataset contains 200 retail product types and 83,739 images, including 53,739 single-product images and 30,000 multi-product images. The product types can be categorized into 17 categories, which contains hierarchical structure information. The training and testing data sets are single product images and checkout images. In single product setting, product images are collected while placing only one product on a turntable. The checkout images are collected from a top view of multiple products placed together. The model needs to be trained on the single product images, but the test is carried out on the checkout images, as shown in Fig. 7. A number of factors are considered in order to provide a real test environment, such as view point, number of objects, and SKU types.

The details of the two datasets are shown in Table 1. Table 2 shows the range of the number of labelled objects contained in a single image in the dataset.

**Table 1:** Statistics of the datasets.

	SKU-110K image numbers	SKU-110K annotated numbers	RPC image numbers	RPC annotated numbers
Training	8219	1208482	6000	73587
Validation	588	90968	24000	294348
Testing	2936	431546	53739	53739
Total	11743	1730996	83739	421674

**Table 2:** Statistics of the annotation in one image.

	Averaged number	Maximum number	Minimum number	PCT995	PCT005
Training	147	476	1	356	61
Validation	154	754	40	582	59

### Metrics

The average precision (AP) based on precision and recall is used to evaluate the performance of the model. The threshold of the mean precision of IoU (Intersection over Union) is 0.5 (mAP@0.5), and that of the IoU threshold is 0.75 (mAP@0.75). We can assess whether the IoU between the predicted box and the real box of each category exceeds the threshold. The precision and recall rate can be calculated under different levels.

After the AP is obtained, the AP of all categories is averaged to obtain the mAP value. The mean value of the results under each threshold value was calculated with $IoU=0.50:0.05:0.95$. The AP value is obtained at 0.05 step intervals within the IoU range of 0.5–0.95.

GFLOPs (Giga floating point of operations) is used as an indicator for computational cost on dense object images. Higher GFLOPs means more efficient model.

### Experimental settings

In this research, the standard ResNet-50 pre-training model on ImageNet is used as the backbone network, and the feature fusion network uses FPN. We deprecate the use of anchor, so there is no necessary to conduct clustering of anchor set in advance, but directly use BRN for regression.

The experiment was conducted on Nvidia RTX3080 GPU running Ubuntu 18.04. In training mode, Adam is used as the optimizer for the proposed BRN. The initial learning rate is 0.001, and the preheating learning rate strategy is adopted. The smaller learning rate is used to preheat learning 1000 steps first, and then the learning rate is adjusted to the specified initial learning rate for exponential decay strategy.

Regarding the hyperparameters in the adaptive focal loss, their values need to be determined based on the proportion of positive and negative samples. The loss function involves two hyperparameters, denoted as $\alpha$ and $\gamma$. Specifically, $\alpha$ serves the purpose of balancing the loss contribution between positive and negative samples, while $\gamma$ functions to downweight relatively simpler samples. The verification results are presented in Table 3, which demonstrate that when employing focal loss ($\alpha \ne 0.5$, $\gamma \ne 0$), there is a substantial increase in the Average Precision (AP) compared to the standard cross-entropy loss. Each row in Table 3 corresponds to one test and there are five tests in total. Especially in the fifth row (the fifth test), we increased $\alpha$ relative to the previous forth test, and decreased $\gamma$ also relative to the previous forth test. Following these findings, it was determined that the optimal values are $\alpha = 0.75$ and $\gamma = 2$. In subsequent experiments, the aforementioned hyperparameter values are adopted as the default choice.

**Table 3:** Model performances under different $\alpha$ and $\gamma$ using SKU-110K dataset.

Model parameters	Parameter $\alpha$	Parameter $\gamma$	mAP
Initial values	0.5	0	48.3
Increased $\alpha$ and $\gamma$	0.75	1	51.6
Increased $\gamma$	0.75	2	52.8
Increased $\gamma$	0.75	3	50.4
Increased $\alpha$ and decreased $\gamma$	0.8	2	51.9

### Model comparisons

**Figure 8:**
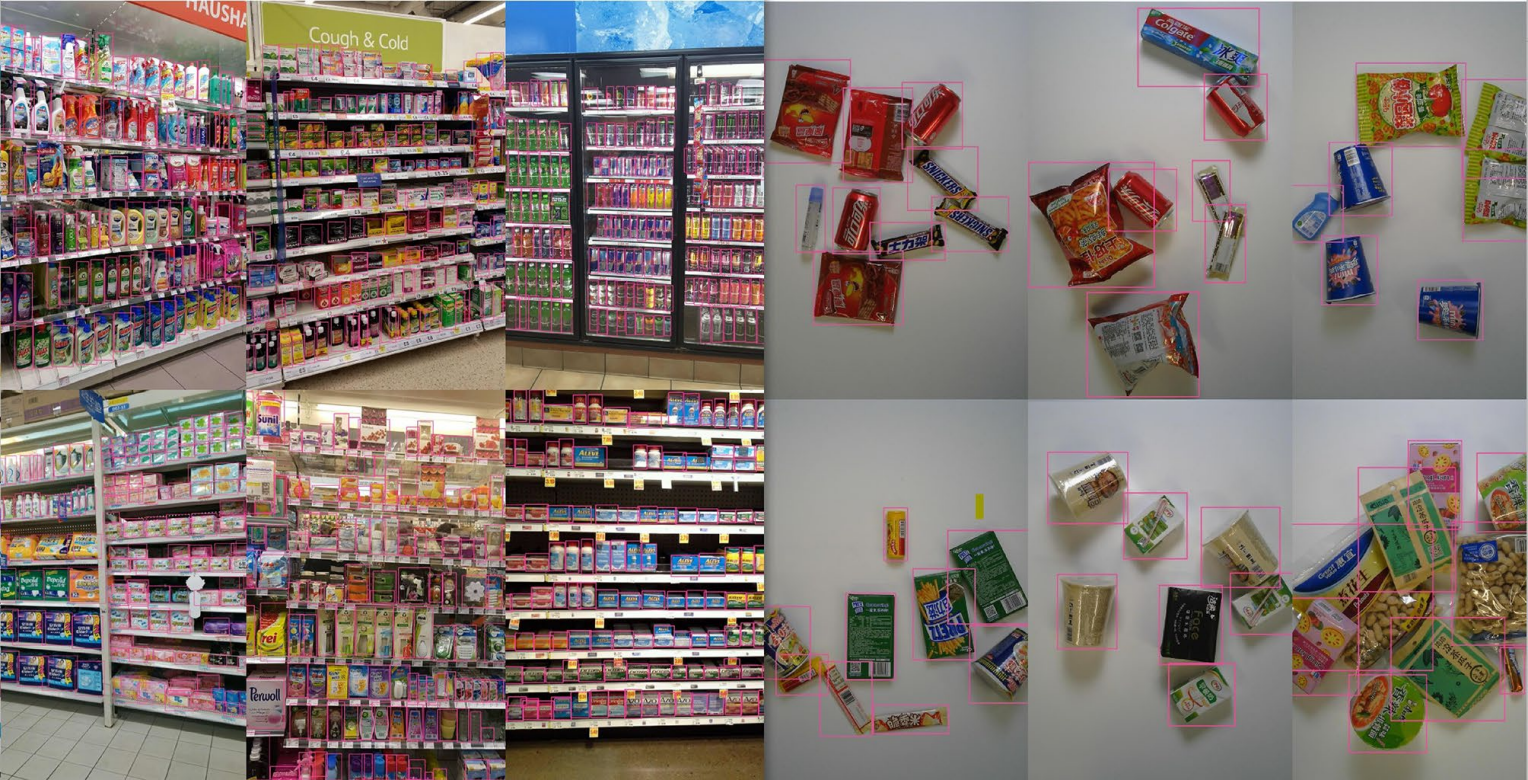
Detection examples of the proposed method on SKU110-K (left: densely placed SKUs) and RPC (right: sparsely placed SKUs).

In order to verify the performance of the model in the product identification scenario, the experiment calculated the mAP of each model on SKU-110K and RPC for comparison. In addition to the model proposed in this paper, other models involved in comparison include Faster-RCNN^11^, VovNet^29^ and RetinaNet^8^. RetinaNet is used as baseline, ResNet is used as backbone network for feature extraction and FPN is used for multi-scale fusion. The boundary regression network (BRN), adaptive focus loss (AFL) and NMS-Score used in this paper are modified based on RetinaNet. Examples of the detection effect of this method on SKU110-K and RPC datasets are shown in Fig. 8. This experiment focuses on comparing the performance differences between our network and RetinaNet. In addition, the experiments also include the widely used target detection network Faster-RCNN as a comparison.

Experimental results for each model are shown in Tables 4 and 5, with the first line showing the performance of our baseline named RetinaNet (ResNet + FPN) achieving a mAP of 37.7 on the SKU-110K dataset. Since the goods on shelves in SKU-110K are too densely packed, and the average number of SKUs in each picture is 154, it is difficult to find the boundary of a single product accurately by using Region Proposal methods such as Faster-RCNN from anchor frame in this scene, mAP is only 4.5.

**Table 4:** Model architectures and results (%) comparison on SKU110k dataset (Hard & Densely placed products).

Model	Backbone	Feature fusion	mAP	AP.75	Parameter size	GFLOPs
RetinaNet	ResNet50	FPN	45.5	48.3	29.8M	95
RetinaNet	ResNet101	FPN	48.2	54.2	48.9M	125.5
Faster-RCNN	ResNet50	FPN	47.1	54.1	37.4M	127.7
Faster-RCNN	ResNet101	FPN	48.2	52.7	56.5M	158.2
Faster-RCNN	VGG16	RPN	42.5	45.5	30.9M	189.9
VovNet	VovNet	N/A	41.2	47.2	35.6M	71.3
YOLO	CSPResNext50	PANet	20.3	28.1	60M	68.3
The proposed model	ResNet50	FPN	52.8	59.8	22.9M	66.5

**Table 5:** Model architectures and results (%) comparison on RPC dataset (Easy & Not Densely placed products).

Model	Backbone	Feature fusion	mAP	AP.75	Parameter size	GFLOPs
RetinaNet	ResNet50	FPN	72.3	74.7	36.3M	182
RetinaNet	ResNet101	FPN	72.1	76.2	55.4M	212.5
Faster-RCNN	ResNet50	FPN	69.8	73.3	41.8M	134.7
Faster-RCNN	ResNet101	FPN	71.8	74.4	59.8M	165.2
Faster-RCNN	VGG16	RPN	70.2	74.1	44.9M	209.4
VovNet	VovNet	N/A	69.3	75.1	35.8M	75.3
YOLO	CSPResNext50	PANet	72.8	75.4	65M	72.4
The proposed model	ResNet50	FPN	73.7	78.1	29.4M	73.1

The densely placed retail objects are challenging, especially when the objects are very similar to each other. Our proposed methods, used modified focal loss function to balanced the samples. NMS score is applied to our experimental comparison, and it is closely related to the actual locations of the densely placed objects. For some cases, the single-stage conventional algorithms perform not ideally. VovNet with its excellent multi-scale feature fusion ability and unique residual connection mode, the mAP is up to 41.2. The proposed method uses boundary regression network to modify the RetinaNet and achieves a 52.8 mAP, which is better than the general target detector. As for the public available RPC dataset, the conventional object detection model has little direct difference due to the small number and loose distribution of targets in RPC dataset.

In order to prove the effectiveness of boundary regression network (BRN), AFL and NMS-Score, our experiment took RetinaNet as Baseline and added the above components respectively for comparison. The experimental results are shown in Table 6.

**Table 6:** Performance improvement using BRN, focal loss, and NMS Score.

Model	mAP (SKU110k)	mAP (RPC)
Baseline	45.5	72.3
Baseline + BRN	48.3	73.4
Baseline + BRN +AFL	49.7	73.8
Baseline + BRN +AFL + NMS Score	52.8	73.7

The model computational efficiency is compared and analyzed. The extensive use of anchor boxes results in increased computational resource requirements. However, our proposed model employs a multi-scale regression process, mitigating the need for such computational expenditures. On the SKU-110K dataset, these algorithm characters led to a considerable improvement in model performance. On the other hand, in the easier scenario, RPC dataset, the products are less densely arranged, the proposed techniques slightly improve the recognition performance. We may conclude that BRN network, AFL and NMS-Score are suitable for densely arranged targets scenarios.

**Figure 9:**
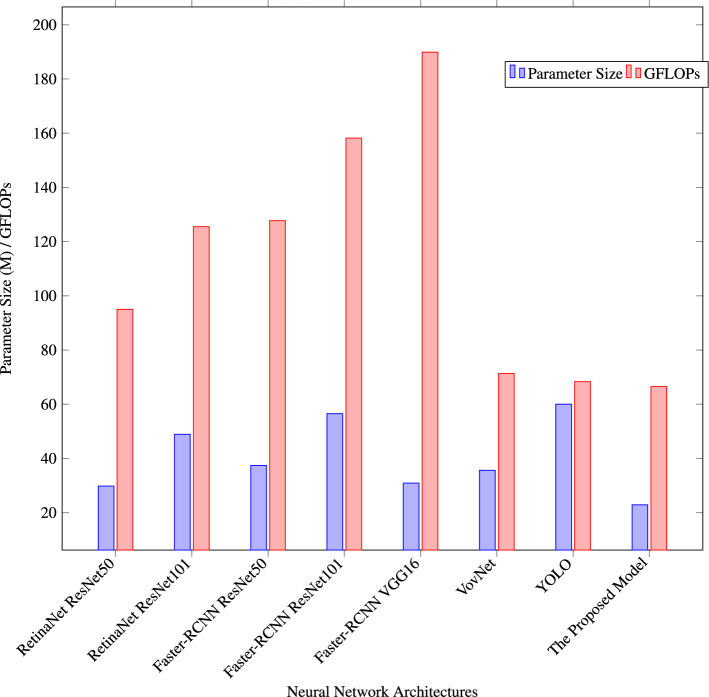
Model efficiency: comparison of parameter size and GFLOPs on SKU110k dataset.

The experimental results depicted in Fig. 9 offer a comprehensive comparison of various neural network architectures concerning their efficiency in terms of parameter size and computational complexity, as measured by GFLOPs, on the SKU110k dataset. The x-axis enumerates the different architectures, including RetinaNet based on ResNet50 and ResNet101, Faster-RCNN based on ResNet50 and ResNet101, Faster-RCNN based on VGG16, VovNet, and the Proposed Model. The y-axis corresponds to the parameter size, and GFLOPs. The bar heights for each architecture represent the respective parameter sizes and computational complexities. Notably, the Proposed Model exhibits remarkable efficiency with the lowest parameter size of 22.9M and a relatively low computational demand of 66.5 GFLOPs, making it a compelling choice for smart retail applications demanding optimal model size and computational efficiency. In contrast, architectures like Faster-RCNN based on ResNet101 and Faster-RCNN based on VGG16 demonstrate higher parameter sizes and computational complexities.

**Figure 10:**
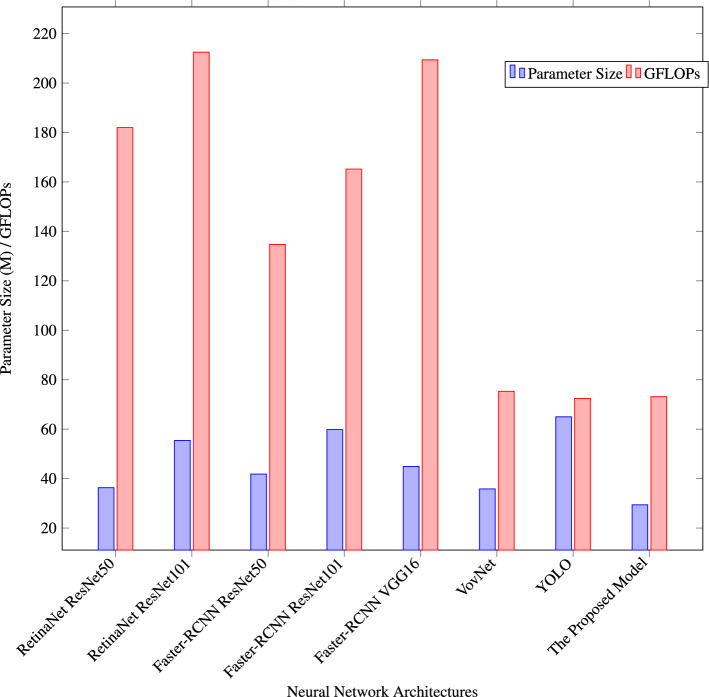
Model efficiency: comparison of parameter size and GFLOPs on RPC dataset.

Using the RPC dataset, as shown in Fig. 10, the bar plot also illustrates a comparative analysis of diverse neural network architectures in terms of their efficiency, evaluated based on both parameter size and computational complexity (measured in GFLOPs). Each architecture is represented on the x-axis, including the same RetinaNet based on ResNet50 and ResNet101, Faster-RCNN based on ResNet50 and ResNet101, Faster-RCNN based on VGG16, VovNet, and the Proposed Model. Notably, the Proposed Model still stands out for its efficiency, with a parameter size of 29.4M and a computational demand of 73.1 GFLOPs. This comparison assists in making selection of proper architecture for cost-sensitive smart retail store applications.

Although, we have achieved considerable improved results on SKUs modeling. Our model, specifically tailored for retail target recognition, does exhibit certain limitations. One constraint is its sensitivity to the specific characteristics of retail objects. Unlike objects in other applications, such as human body detection, retail items typically possess well-defined and factory-manufactured appearances. Our model may not be as well-suited for scenarios where objects undergo dynamic changes over time.

Furthermore, the emphasis on capturing subtle differences between similar SKUs in retail introduces a limitation when attempting to generalize the model to vastly different scenes. The fine-grained distinctiveness modeled for retail scenarios may result in reduced robustness when confronted with diverse scenes, such as the classification of biological species categories. In these cases, the model should ideally exhibit robustness to subtle differences in visual characteristics. Achieving this balance between specificity for retail scenarios and the capacity to generalize across a wide range of scenes is an ongoing challenge in refining our model’s performance.

## Conclusion

In this paper, a high performance dense retail object detector based on RetinaNet is designed for some problems involved in retail product detection. The detector consists of backbone network, feature fusion network and boundary regression network. Boundary regression is a universal computer vision problem, that can be applied to many visual understanding applications. The backbone network uses pre-trained ResNet50 to achieve feature extraction, and the results are input into FPN, and then the features fused by FPN are sent into BRN to obtain the precise position of the bounding box and NMS-Score. In addition, AFL is introduced to solve the imbalance between positive and negative samples as well as difficult and easy samples. Meanwhile, the NMS strategy of the end is improved, and the NMS-Score returned from the network is used as the basis of NMS ranking. Experiments show that the boundary regression network proposed in this paper can significantly improve the detection ability of dense objects, and the new loss function introduced can improve mAP to a certain extent, while NMS Score contributes to the accuracy of the final bounding box.

## Data Availability

The datasets generated and analysed during the current study are available in two databases: SKU-110K dataset, https://www.kaggle.com/datasets/thedatasith/sku110k-annotations and RPC dataset https://rpc-dataset.github.io/.
